# Changes in Antioxidant Enzymes Activity and Metabolomic Profiles in the Guts of Honey Bee (*Apis mellifera*) Larvae Infected with *Ascosphaera apis*

**DOI:** 10.3390/insects11070419

**Published:** 2020-07-06

**Authors:** Zhiguo Li, Mengshang Hou, Yuanmei Qiu, Bian Zhao, Hongyi Nie, Songkun Su

**Affiliations:** College of Animal Sciences (College of Bee Science), Fujian Agriculture and Forestry University, Fuzhou 350002, China; zhiguo.li@fafu.edu.cn (Z.L.); hhoums@sina.com (M.H.); ym.qiu@icloud.com (Y.Q.); zba123@outlook.com (B.Z.); hnhynie@126.com (H.N.)

**Keywords:** *Apis mellifera*, chalkbrood, *Ascosphaera apis*, metabolomics, enzyme activities

## Abstract

The fungus *Ascosphaera apis*, an obligate fungal pathogen of honey bee brood, causes chalkbrood disease in honey bee larvae worldwide. Biological characteristics of the fungal pathogen and the molecular interactions between *A. apis* and honey bees have been studied extensively. However, little is known about the effects of *A. apis* infection on antioxidant enzyme activities and metabolic profiles of the gut of honey bee larvae. In this study, sandwich enzyme-linked immunosorbent assay and LC-MS based untargeted metabolomic analysis were employed to determine the changes in the specific activities of antioxidant enzymes and the metabolomic profiles in gut tissues of *A. apis*-infected larvae (10^5^ *A. apis* spores per larva) and controls. Results showed that specific activities of superoxide dismutase, catalase and glutathione S-transferase were significantly higher in the guts of the control larvae than in the guts of the *A. apis*-infected larvae. The metabolomic data revealed that levels of 28 and 52 metabolites were significantly higher and lower, respectively, in the guts of *A. apis*-infected larvae than in the guts of control larvae. The 5-oxo-ETE level in the infected larvae was two times higher than that in the control larvae. Elevated 5-oxo-ETE levels may act as a potential metabolic biomarker for chalkbrood disease diagnosis, suggesting that *A. apis* infection induced obvious oxidative stress in the honey bee larvae. The levels of metabolites such as taurine, docosahexaenoic acid, and L-carnitine involved in combating oxidative stress were significantly decreased in the gut of *A. apis*-infected larvae. Overall, our results suggest that *A. apis* infection may compromise the ability of infected larvae to cope with oxidative stress, providing new insight into changing patterns of physiological responses to *A. apis* infection in honey bee larvae by concurrent use of conventional biochemical assays and untargeted metabolomics.

## 1. Introduction

As one of the most important managed pollinators, the Western honey bee (*Apis mellifera*) is responsible for the pollination of crops worth $215 billion worldwide [1]. Therefore, the different threats to honey bee health caused by abiotic and biotic stresses have always been a major concern, and the combinations of parasites, pathogens, pesticides, and habitat loss could contribute to honey bee colony losses [1,2].

The fungus *Ascosphaera apis*, an obligate fungal pathogen of honey bee brood, causes chalkbrood disease in honey bee larvae [3]. Larvae aged 3–4 days are most susceptible to *A. apis* infection through the ingestion of food containing fungal spores delivered by contaminated nurse bees [4]. *A. apis* infection damages the gut lining of the host, and the fungal hyphae penetrate into the gut wall of the infected larva [5]. Transcriptomic studies of *A. apis* indicated that fungal transcripts encoding chitinases may contribute to the penetration of the larval gut during host invasion by *A. apis* [6]. *A. apis* infection not only acts as a direct disease stressor causing chalkbrood in honey bees, but also interacts with other biotic and abiotic stressors. Worker honey bees from chalkbrood-infected colonies exhibit significantly elevated deformed wing virus (DWV) viral load [7]. Common honey bee viruses, such as DWV, could infect and replicate in *A. apis* [8]. More severe symptoms can be found in *A. apis*-infected larvae, because the virulence of *A. apis* is likely to increase due to chilling stress [9].

The genes responsible for regulating oxidative stress response in honey bee larvae are probably involved in combating pathological tissue damage induced by *A. apis* infection [4,10]. Oxidative damage caused by reactive oxygen species (ROS) has been linked with aging, behavioral dysfunction and cell death in organisms [11,12]. Catalase (CAT), glutathione S-transferase (GST), and superoxide dismutase (SOD) are the three major ROS scavenging and antioxidant enzymes in honey bees; they play important roles in antioxidant defense in honey bees exposed to biotic and abiotic stressors [12]. Honey bees from lead-contaminated industrial areas have lower levels of CAT activities than those from unpolluted areas [13,14], and the CAT activities in bees exposed to 0.001 mgL^−1^ of CdCl_2_ are significantly lower than those in control bees [14]. These three antioxidant enzymes can protect a stored sperm against oxidative damage in the spermatheca of mated queens [15]. Honey bee feeding diets containing high levels of protein have high levels of mRNA encoding CAT, GST, and SOD during the larval stage and a lengthened lifespan after emergence, indicating that elevated expression levels of antioxidant enzymes positively affect the longevity of honey bees [16]. 

The biological characteristics of the fungal pathogen and the molecular interactions between *A. apis* and honey bees have been studied extensively. However, little is known about the effects of *A. apis* infection on the metabolic profiles of the gut of honey bee larvae. As an important complement to transcriptomic studies, liquid chromatography–mass spectrometry (LC–MS)-based metabolomics has been widely employed in identifying and quantifying metabolites related to organisms exposed to various biotic and abiotic stressors [17]. Both ROS scavenging enzymes and small non-enzymatic molecules that contribute to maintain the redox balance of cells are involved in antioxidant defense in organisms [18]. Whether the three major antioxidant enzymes and small non-enzymatic molecules play roles during the process of *A. apis* infection remains unknown. Thus, in the present study, ultra-high performance liquid chromatography coupled with a high-resolution mass spectrometer (UHPLC-HRMS) was used to determine the differential metabolites in the gut tissues of *A. apis*-infected larvae and the controls. Sandwich enzyme-linked immunosorbent assay (ELISA) was conducted to determine the changes in the specific activities of the antioxidant enzymes in *A. apis*-infected larvae. The findings showed that *A. apis* infection induced oxidative stress in honey bee larvae and fungal infection may compromise the antioxidant defensive ability of the larvae.

## 2. Materials and Methods 

### 2.1. Honey Bee Larvae Inoculated with A. apis

Small pieces of chalkbrood mummies were surface-sterilized and incubated in MY-20 medium in accordance with the methods described in previous studies [5,8]. *A. apis* spores were obtained and purified as described previously [5], and fungal spore was counted using a hemocytometer as described by Human et al. [19]. A subsample of the purified spores was used for DNA extraction using a DNAzol reagent (Invitrogen, Carlsbad, CA, USA), and the purified DNA and previous published primer pair 3-F1 and 3-R1 were used to confirm the species status of *A. apis* through PCR analysis [20].

Three different apparently healthy colonies were used in this study. To obtain larvae of the same age, the queen of each colony was confined to a comb with empty worker cells using a queen excluder cage for six hours. After egg hatched, the 2 day-old larvae were transferred from three different combs to a 48-well microtiter plate with one larva per well, and 12 microtiter plates were used and placed in an incubator (34.5 °C, 95% RH) in complete darkness. Artificial diets were prepared on the basis of the recipe described in previous studies [21], and the larvae were fed individually once daily. The 2 day-old larvae were each fed with 50 μL of the diet. The 3 day-old larvae were randomly divided into two groups with six plates in each group. One group of 3 day-old larvae was used as the control group and fed with 50 μL of the original diet, and another group of 3 day-old larvae was used as the treatment group and fed with 50 μL of the treated diet containing 10^5^ fungal spores. The original diet was fed to the 4 and 5 day-old larvae from the two groups, and each larva was fed with 100 μL of the diet. Six plates containing 6 day-old larvae (three plates per group) were collected. The whole gut was dissected from the larvae with sterile forceps, and the isolated individual whole gut was frozen immediately in liquid nitrogen and then stored at −80 °C for later use. The *A. apis* infection status of the gut samples was confirmed via PCR. The remaining six plates of larvae (three plates per group) were transferred to new 48-well microtiter plates after defecation and placed in the incubator for three days to monitor the fungal infection rate.

### 2.2. Enzyme Activity Analysis

The specific activities of antioxidant enzymes CAT, GST, and SOD were determined in accordance with our previous work [22,23]. In brief, guts from three larvae were dissected and pooled for each group, and the six pooled samples from 18 larval guts were analyzed. Total proteins were extracted from each pooled sample using a total protein extraction kit following the manufacturer’s protocol (TransGen Biotech, Beijing, China). Each pooled gut sample was twice rinsed with precooled 1 mL phosphate-buffered saline, followed by centrifugation at 500× *g* for 5 min at 4 °C. The supernatant was discarded and 1 mL of total protein extraction buffer was added to each sample, followed by homogenization using a glass homogenizer. The homogenized suspension was transferred to another 1.5 mL tube and incubated on ice for 30 min by vibrating the tube every 10 min during this period. The sample was then centrifuged at 14,000× *g* for 10 min at 4 °C, and the supernatant containing total proteins was collected carefully. The extracted proteins were quantified via bicinchoninic acid assay. The activities of CAT, GST, and SOD were analyzed using the total proteins extracted by sandwich ELISA kit in accordance with the company’s instructions (mlbio, Shanghai, China). Three antibodies (anti-SOD, anti-CAT, and anti-GST) were used, and the microtiter wells were precoated with the antibody respectively. A total of 50 μL of total proteins from each sample or standard enzymes were added into the wells of the antibody precoated microtiter plate. Then, 100 μL of the corresponding antibody conjugated with horseradish peroxidase was added into each reaction well. Incubate the microtiter plate at 37 °C for 30 min. The reaction solutions were then discarded and the reaction well was washed five times using wash buffer. The chromogenic reaction was carried out at 37 °C for 60 min after 100 μL of tetramethylbenzidine was added into the reaction well. For each reaction well, the optical density at 450 nm was measured, and the activity of each antioxidant enzyme was determined according to the standard curve generated by diluting standard enzymes of known activities. The specific activity of each antioxidant enzyme was expressed as units of enzyme activity per milligram of protein [24]. 

### 2.3. Chemicals and Extraction of Metabolites

Methanol, acetonitrile, ammonium acetate and ammonium hydroxide were obtained from CNW Technologies GmbH (Düsseldorf, Germany), and all four solvents were of LC-MS grade. 2-Chloro-l-phenylalanine (>99%) was obtained from Hengbai Biotech Co., Ltd. (Shanghai, China) and acted as an internal standard.

A mixture of methanol, acetonitrile, and water (2:2:1, *v*/*v*) was used as an extraction solvent for the initial extraction. Each sample consisted of four guts, and 10 samples were used for each group. Each sample was placed in an Eppendorf tube, and 1 mL of extraction solvent containing the internal standard (2 μg/mL) was added to each tube and vortexed for 30 s. The mixture was then homogenized in a ball mill for 4 min at 45 Hz, followed by ultrasound treatment for 5 min via incubation in ice water bath. The homogenization process was repeated two times. After homogenization, the mixture was incubated for 1 h at −20 °C. The supernatant was collected after the mixture was centrifuged at 12,000 rpm for 15 min at 4 °C and then evaporated in a vacuum concentrator. The dried extracts were reconstituted with 200 μL of extraction solvent consisting of acetonitrile and water (1:1, *v*/*v*). The solutions were vortexed for 30 s and sonicated for 10 min at 4 °C in an ice water bath. The sonicated solutions were centrifuged at 12,000 rpm for 15 min at 4 °C, and 75 μL of the supernatant was transferred into a 2 mL autosampler vial for UHPLC-QTOF-MS analysis. In addition, 10 ul of the supernatant from each sample was pooled, and 75 μL of the pooled supernatant was used as quality control (QC) samples for UHPLC-QTOF-MS analysis. 

### 2.4. UHPLC-QTOF-MS Analysis

UHPLC-QTOF-MS analyses were conducted on the UHPLC system (Agilent 1290, Agilent Technologies, Santa Clara, CA, USA) with UPLC BEH Amide column (1.7 μm, 2.1 mm × 100 mm, Waters, Milford, CT, USA) coupled to the AB Sciex Triple TOF 6600 system (Framingham, MA, USA). The mobile phase was prepared using two solvents. Solvent A consisted of 25 mM of NH_4_Ac and 25 mM of NH_4_OH in water (pH = 9.75), and solvent B was acetonitrile. Gradient elution was carried out as follows: 0 min, 95% B; 0.5 min, 95% B; 7 min, 65% B; 8 min, 40% B; 9 min, 40% B; 9.1 min, 95% B; and 12 min, 95% B. The injection volume for all samples was 1 μL, and the flow rate of the mobile phase was 0.5 mL min^−1^. The Triple TOF mass spectrometer assisted by information-dependent acquisition method was used to collect MS/MS spectra. The MS data were acquired via Analyst TF 1.7 software (AB Sciex, Framingham, MA, USA). During data collection, 12 precursor ions with an intensity of more than 100 were selected for fragmentation at a collision energy of 30 V (15 MS/MS events at an ion accumulation time of 50 ms each). The instrumental parameters of the electrospray ionization source were set as follows: ion source gas 1 = 60 Psi; ion source gas 2 = 60 psi; curtain gas = 35 psi; source temperature = 600 °C; and ion spray voltage floating = 5000 V and −4000 V for positive and negative modes, respectively.

### 2.5. Statistical Analyses

Data were collected in positive and negative ion modes. ProteoWizard was used to convert MS raw data (wiff files) into mzXML format [25]. The dataset was analyzed on R package XCMS version 3.2 to generate a data matrix consisting of retention time, mass-to-charge ratio, and peak intensity [26]. Afterward, peak annotation and metabolite identification against an in-house MS2 database were performed on R package CAMERA [27]. The average abundance of each metabolite in ten biological replicates was calculated for each group, and the fold change is the ratio of the average abundance of each metabolite in the *A. apis*-infected group to the control group. The ratios were then subjected to log2 transformation to simplify data presentation. Multivariate analysis using orthogonal partial least squares discriminant analysis (OPLS-DA) on the software SIMCA version 15.0.2 (Umea, Sweden) was employed to explore the dissimilarity and classification between the two groups. Variable importance in the projection (VIP) was obtained on the basis of the OPLS-DA model, and the metabolites were considered significantly differential if they had a *p* value < 0.05 (Student’s *t* test) and a VIP value > 1. The pathways associated with differential metabolites were identified using MetaboAnalyst 4.0 (https://www.metaboanalyst.ca/) and KEGG (https://www.genome.jp/kegg/). Student’s *t* test was performed on SPSS 18.0 to compare the differences in enzyme activities between the two groups.

## 3. Results

### 3.1. Rates of Chalkbrood Symptoms

All the gut samples of the *A. apis*-infected larvae (n = 9) were positively infected with *A. apis* as revealed by PCR test, whereas those of the control larvae (n = 9) were negative for *A. apis* infection (Figure S1). Most of the *A. apis*-infected larvae developed typical chalkbrood symptoms, and the percentage of 9 day-old pupae covered with white, cotton-like mycelium was 76% (n = 130). All the control larvae developed well in the pupal stage without any obvious disease symptoms (n = 144) (Figure S2). Significant differences in the rates of chalkbrood symptoms were observed between the two larvae groups (*p* < 0.0001, Fisher’s exact test).

### 3.2. Effects of A. apis Infection on Gut Enzyme Activities

**Figure 1:**
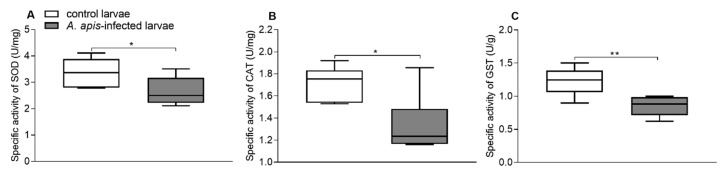
Comparative analysis of specific activities of SOD (superoxide dismutase, (**A**)), CAT (catalase, (**B**)), and GST (glutathione S-transferase, (**C**)) between *A. apis*-infected larvae and controls. The error bar indicates SD; Asterisks represent significant differences (Student’s *t* test, * *p* < 0.05; ** *p* < 0.01).

Significant differences in antioxidant enzyme levels were found between the control and the *A. apis*-infected larvae. The SOD activities in the guts of the control and *A. apis*-infected larvae were 3.37 ± 0.53 and 2.66 ± 0.54 U/mg, respectively (Figure 1A). The CAT activities in the guts of the control and *A. apis*-infected larvae were 1.72 ± 0.15 and 1.33 ± 0.27 U/mg, respectively (Figure 1B), and their GST activities were 1.23 ± 0.22 and 0.85 ± 0.15 U/g, respectively (Figure 1C). The specific activities of all three enzymes were significantly higher in the guts of the control larvae than in the guts of *A. apis*-infected larvae (for SOD, *t*-test: *t* = 2.322, df = 10, *p* = 0.043; for CAT, *t*-test: *t* = 3.021, df = 10, *p* = 0.013; for GST, *t*-test: *t* = 3.487, df = 10, *p* = 0.006).

### 3.3. Analysis of Significantly Differential Metabolites

**Figure 2:**
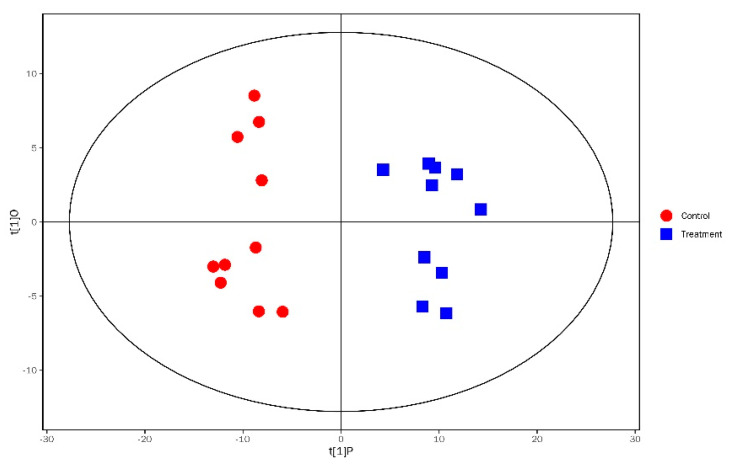
The OPLS-DA model for assessing group differences in the metabolite profiles between *A. apis*-infected larvae (blue square) and controls (red dot).

**Figure 3:**
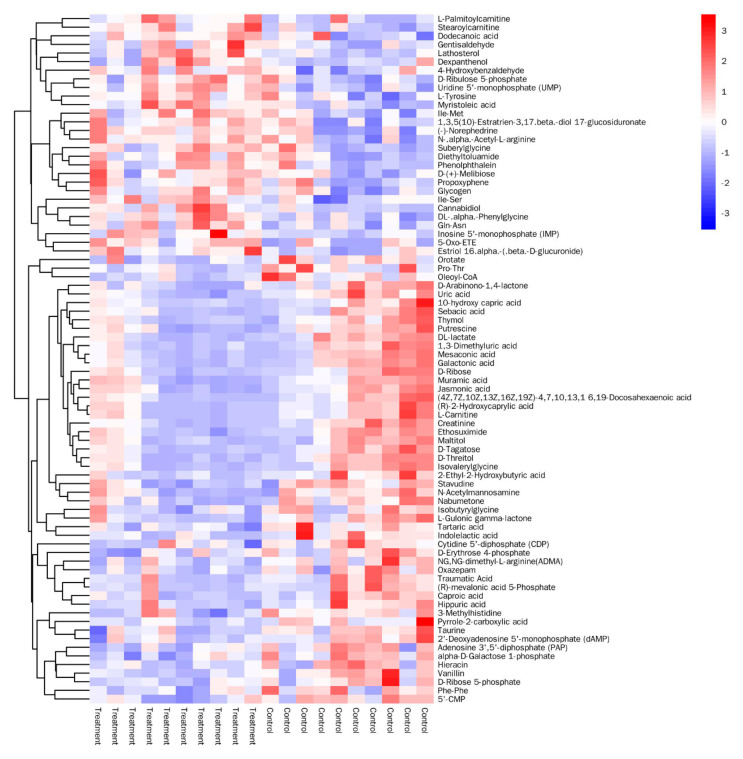
Heat map visualization for significantly differential metabolites identified in gut tissues between *A. apis*-infected larvae and controls. Colors indicate the relative level of metabolites in the samples, ranging from highest (red) to lowest (blue): red indicates a relatively higher level, blue indicates a relatively lower level and white indicates the relative level of metabolites falls between high and low.

OPLS–DA analysis was performed to assess the group differences between the two types of gut samples. The R2Y and Q2 values were 0.946 and 0.598, respectively, and the results of OPLS–DA analysis indicated that the *A. apis*-infected larvae were distinctly separated from the healthy controls (Figure 2). On the basis of the VIP value derived from the OPLS–DA model (VIP > 1) and *p* < 0.05, a total of 80 unique significantly differential metabolites were identified in the two groups (Figure 3). The levels of 28 and 52 metabolites were significantly higher and lower, respectively, in the guts of the *A. apis*-infected larvae than in the guts of the control larvae (Table S1). Table 1 lists 30 significantly differential metabolites with the highest fold difference.

### 3.4. Pathway Enrichment Analysis of Significantly Differential Metabolites

**Figure 4:**
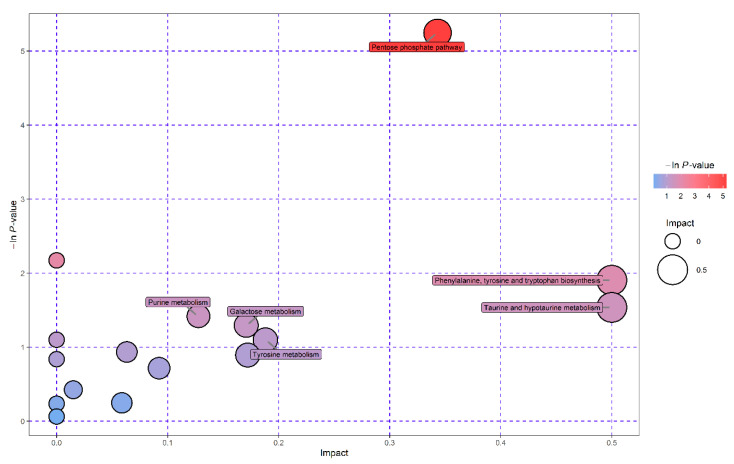
The bubble plot displays significant metabolic pathways of the mapped differential metabolites between *A. apis*-infected larvae and controls. Pathway impact score is proportional to the bubble size, and significance level of the pathway is denoted by the bubble color, ranging from highest (red) to lowest (blue).

Pathway enrichment analysis was conducted on the significantly differential metabolites in the *A. apis*-infected and control larvae by using MetaboAnalyst 4.0 to identify the metabolic pathway related to *A. apis* infection in the guts of honeybee larvae. A total of 18 metabolites with significantly altered levels were mapped to 16 KEGG pathways, and the levels of six and 12 metabolites were significantly higher and lower, respectively, in the guts of the *A. apis*-infected larvae than in the guts of the control larvae. A set of six most relevant pathways was selected based on –ln *p*-value >1 and pathway impact score >0.01 (Table S2). The identified pathways were pentose phosphate pathway, taurine and hypotaurine metabolism, purine metabolism, galactose metabolism, tyrosine metabolism and phenylalanine, tyrosine and tryptophan biosynthesis (Figure 4). 

## 4. Discussion

*A. apis* infection probably cause oxidative stress in infected larvae, and the genes involved in combating oxidative stress play functional roles associated with chalkbrood infection [4]. Transcriptional profiling of honey bee larvae infected with *A. apis* revealed that the expression levels of *vitellogenin* and *hexamerins* are significantly down-regulated in *A. apis*-infected larvae [10], and the activities of both were positively correlated with antioxidant capacity in honey bees [11]. The increase in SOD enzymatic activity promotes oxidative stress resistance and is closely associated with extended lifespan in *Drosophila melanogaster*; the increase in CAT activities indicate upregulation of cellular defenses in resisting oxidative damage in *Drosophila melanogaster* [28]. GST is a multifunctional enzyme that detoxifies endogenous and exogenous compounds and is associated with oxidative stress response in the larval midgut of *Drosophila melanogaster* [29]. Thus, *A. apis* infection may decrease the antioxidant capacity of honey bee larvae. In the present study, the CAT, GST, and SOD in the guts of *A. apis*-infected larvae exhibited significantly decreased specific activities compared with those in the guts of control larvae. The decreased activities of all three antioxidant enzymes provided a direct evidence that *A. apis* infection decreased the antioxidant capacity of honey bee larvae. In addition, previous studies have shown that abiotic stressors, such as chemicals and nutrition, could decrease the activities and transcript levels of the antioxidant enzyme in honey bees [13,14,16]. Therefore, biotic and abiotic stressors may diminish the ability of honey bees to cope with oxidative stress.

Previous studies have demonstrated that 5-oxo-ETE is synthesized in large amounts when organisms are exposed to oxidative stress [30,31]. In the present study, the 5-oxo-ETE levels of *A. apis*-infected larvae were two times higher than those of the control larvae, suggesting that *A. apis* infection induces obvious oxidative stress in honey bee larvae and 5-oxo-ETE may act as a potential metabolic biomarker for the diagnosis of chalkbrood disease. Furthermore, the levels of metabolites involved in combating oxidative stress were significantly decreased in the gut of *A. apis*-infected larvae. The *A. apis*-infected larvae exhibited significantly lower levels of taurine than the control larvae. Taurine, an amino acid, is widely distributed in insect and animal tissues and has antioxidant effects against oxidative stress in rats induced by lead [32,33]. In addition, the d-ribose level was more than two times lower in the *A. apis*-infected larvae than in the control larvae in the present study. Previous studies have indicated that d-ribose plays an essential role in ROS neutralization and benefits cells during oxidative stress [34]. Significantly decreased concentrations of l-carnitine, which exhibits distinct antioxidant activities [35,36], were also found in *A. apis*-infected larvae. Varied levels of docosahexaenoic acid have been found in insects [37,38], and the protective effects of docosahexaenoic acid against oxidative stress were reported in previous studies [39]. In the present study, the docosahexaenoic acid level was nearly three times lower in the *A. apis*-infected larvae than in the control larvae, suggesting that the antioxidant capacity of the *A. apis*-infected larvae may be repressed. Previous studies have shown that uric acid could scavenge oxyradicals during larval development in insects [40]. In the present study, the uric acid level was significantly decreased in the guts of the *A. apis*-infected larvae, indicating that *A. apis* infection may compromise the ability of infected larvae to cope with oxidative stress. From a metabolic perspective, the results further indicated that *A. apis* infection induced oxidative stress in the honey bee larvae and decreased levels of metabolites involved in combating oxidative stress could compromise the antioxidant defenses of the infected larvae. In addition, the glycogen level was significantly higher in the *A. apis*-infected larvae than that in the control larvae. Glycogen is the main fuel used by honey bees in response to stressful activities [41]. High glycogen levels indicated that *A. apis*-infected larvae may accelerate the metabolism of energy reserves in responding to pathogen-induced energetic stress. It should also be noted that muramic acid is a compound of bacterial origin and can be effectively used as a bacterial biomarker in microbial communities [42]. The concentration of muramic acid was significantly reduced in the guts of the *A. apis*-infected larvae, indicating that *A. apis* infection may disturb bacterial communities which may eventually result in metabolic imbalances in *A. apis*-infected larvae [43]. Further research is needed to investigate the interaction among symbiotic microbes, *A. apis*, and honey bee larvae. In addition, dose-dependent physiological and immune responses were found in *Galleria mellonella* larvae inoculated with different levels of β-glucan [44], and honey bees infected with different spore doses of *Nosema ceranae* exhibited differential immune responses [45,46]. Jensen et al. clearly demonstrated dose-dependent effects of *A. apis* infection on the development of infection in *A. mellifera* larvae [47], and different spore doses of the fungus *A. apis* may lead to differential physiological responses in the infected larvae. Further studies are needed to examine the changing patterns of metabolic and immune responses in larvae exposed to different spore doses of *A. apis* at different time points after infection.

Metabolic pathway enrichment analysis indicated that four significantly differential metabolites were mapped to the pentose phosphate pathway (PPP), which primarily contributes to host cell defense against oxidative stress and helps in tissue growth and development [18,48]. Reduced nicotinamide adenine dinucleotide phosphate (NADPH) generated in the PPP acts as a cofactor for antioxidant enzymes in organisms during oxidative stress conditions [48]. As glutathione (GSH)-dependent enzyme, GST protects the cell against oxidative damage via quenching of the ROS with the addition of GSH [49]. NADPH plays essential roles in continuous replenishment of GSH by acting as glutathione reductase cofactor and additionally protects CAT activities from H_2_O_2_ inactivation [48]. The PPP and SOD play essential overlapping roles in oxidative damage protection given that NADPH relieves the methionine auxotrophic phenotype of *Saccharomyces cerevisiae* with SOD1 gene mutation [50]. The levels of three differential metabolites (d-ribose 5-phosphate, d-ribose, and d-erythrose 4-phosphate) mapped to the pathway were significantly lower in the *A. apis*-infected larvae, indicating a possible dysregulation of the PPP and unbalanced high ROS levels in the *A. apis*-infected larvae. The PPP dysregulation not only affected its own roles in maintaining the redox balance of cells but may also have adverse effects on activities of the three antioxidant enzymes. Taurine was the only one metabolite mapped to taurine and hypotaurine metabolism pathway and significantly lower levels of taurine were found in *A. apis*-infected larvae. It has been suggested that taurine and hypotaurine metabolism plays an antioxidant function in organisms [51]. *A. apis* infection may compromise the antioxidant defenses of larvae by downregulating the levels of metabolites related to the two metabolic pathways. Furthermore, three (d-ribose 5-phosphate, 2′-deoxyadenosine 5′-monophosphate, and uric acid) of the four significantly differential metabolites mapped to purine metabolism were significantly decreased in the *A. apis*-infected larvae. The disorders of this metabolism could lead to a deficient immune system in the infected larvae, thus making them more susceptible to infection from other stressors, including viruses [7,52]. The level of l-tyrosine was significantly higher in the *A. apis*-infected larvae, and l-tyrosine was mapped to phenylalanine, tyrosine and tryptophan biosynthesis pathway and tyrosine metabolism pathway. The normal levels of amino acids associated with these two pathways play key roles in sclerotization of the cuticle and synthesis of pigment compounds during insect development [53,54]. The disorder of the two pathways may affect the physiological and mechanical properties of the cuticle of honey bee larvae infected with *A. apis*. The levels of melibiose and galactose-1-phosphate were significantly higher and lower in the *A. apis*-infected larvae, respectively. The two metabolites were mapped to galactose metabolism which is closely related to energy metabolism and plays structural roles for biosynthesizing macromolecules in organisms [55]. Disorders of galactose metabolism may affect larval growth and development. 

## 5. Conclusions

Taken together, the results indicated that honey bee larvae were subjected to increased oxidative stress due to *A. apis* infection. The specific activities of antioxidant enzymes and levels of metabolites involved in combating oxidative stress were significantly decreased in the gut of honey bee larvae exposed to a high spore dose of *A. apis*. Unbalanced high ROS levels possibly induced oxidative stress-related abnormal development and immunodeficiency in *A. apis*-infected larvae. 

## Figures and Tables

**Table 1:** A list of 30 significant differential metabolites with the highest fold difference in guts between *A. apis*-infected larvae and controls with VIP > 1 and *p* < 0.05.

Metabolites	HMDB ID	VIP Value	*p* Value	log2 Fold-Change
10-hydroxy capric acid	/	2.221866	0.040449	−1.583220998
Docosahexaenoic acid	HMDB02183	2.348188	0.018642	−1.400723937
DL-lactate	HMDB01311	2.32267	0.001646	−1.383585277
L-Carnitine	HMDB00062	2.366084	0.019981	−1.249241729
(R)-2-Hydroxycaprylic acid	HMDB0002264	2.320383	0.027084	−1.238792434
D-Threitol	HMDB04136	2.359414	0.001944	−1.17578676
Ethosuximide	HMDB14731	1.948189	0.007349	−1.162173255
D-Ribose	HMDB00283	2.181031	0.015077	−1.158423407
2-Ethyl-2-Hydroxybutyric acid	HMDB01975	2.233223	0.017102	−1.157991419
Isovalerylglycine	HMDB00678	2.120458	0.008138	−1.156552634
(R)-mevalonic acid 5-Phosphate	HMDB01343	2.030673	0.02835	−1.145439547
D-Tagatose	HMDB03418	2.313209	0.009622	−1.14421462
Sebacic acid	HMDB00792	2.30288	0.017322	−1.068341762
5-Oxo-ETE	HMDB0010217	1.463255	0.01796	1.059981866
Traumatic Acid	HMDB00933	2.371702	0.02379	−0.994244091
Pro-Thr	HMDB0029027	1.823645	0.027625	−0.954262735
Cannabidiol	HMDB0014613	2.030682	0.008316	0.923946494
Pyrrole-2-carboxylic acid	HMDB04230	2.205492	0.041314	−0.900458683
1,3-Dimethyluric acid	HMDB01857	2.190309	0.008027	−0.897637652
Muramic acid	HMDB0003254	2.362325	0.019607	−0.890529844
Galactonic acid	HMDB00565	2.221866	0.003087	−0.883735874
Lathosterol	HMDB01170	1.710821	0.018031	0.876832978
Indolelactic acid	HMDB00671	2.053832	0.01721	−0.845847707
Jasmonic acid	HMDB32797	2.261791	0.024457	−0.822748259
Hippuric acid	HMDB00714	2.139678	0.04853	−0.813266898
Creatinine	HMDB00562	2.372534	0.005277	−0.781944734
Stearoylcarnitine	HMDB00848	2.005948	0.004388	0.780781325
Gln-Asn	HMDB0028792	1.732313	0.004701	0.778386048
Maltitol	HMDB0002928	1.773791	0.023112	−0.774603717
5′-CMP	HMDB0000095	1.934189	0.003266	−0.762506922

## Supplementary Materials

The following are available online at https://www.mdpi.com/2075-4450/11/7/419/s1, Figure S1: Detection of *A. apis* in the gut sample from *A. apis*-infected larvae and controls by PCR test at 3 days post-infection. Figure S2: All the control larvae developed well without any obvious disease symptoms (A); the Apis-infected larvae developed typical chalkbrood symptoms at 6 days post-infection (B). Table S1: A total of 80 unique significantly differential metabolites were identified between *A. apis*-infected larvae and controls. Table S2: The six most relevant pathways were selected based on –ln *p*-value > 1 and pathway impact score > 0.01.
