# Supplementary material for: Changes in Antioxidant Enzymes Activity and Metabolomic Profiles in the Guts of Honey Bee (Apis mellifera) Larvae Infected with Ascosphaera apis
Source: Insects. 2020 Jul 6;11(7):419. doi: 10.3390/insects11070419 (PMC7412215; doi:10.3390/insects11070419)
Supplement: Supplementary file 1 [file insects-11-00419-s001.zip › Supplementary Files/Captions for Figures S1, S2, Tables S1, S2.docx]

**Figure S1.** Detection of *A. apis* in the gut sample from *A. apis*-infected larvae and controls by PCR test at 3 days post-infection.

**Figure S2.** All the control larvae developed well without any obvious disease symptoms (A); the *Apis*-infected larvae developed typical chalkbrood symptoms at 6 days post-infection (B), and the percentage of larvae covered with white, cotton-like mycelium was 77% (37/48) in this 48-well microtiter plate.

**Table S1.** A total of 80 unique significantly differential metabolites were identified between *A. apis*-infected larvae and controls.

**Table S2.** The six most relevant pathways were selected based on –ln P-value > 1 and pathway impact score > 0.01.
