# Supplementary material for: Changes in Antioxidant Enzymes Activity and Metabolomic Profiles in the Guts of Honey Bee (Apis mellifera) Larvae Infected with Ascosphaera apis
Source: Insects. 2020 Jul 6;11(7):419. doi: 10.3390/insects11070419 (PMC7412215; doi:10.3390/insects11070419)
Supplement: Supplementary file 1 [file insects-11-00419-s001.zip › Supplementary Files/Figure S1.pdf]

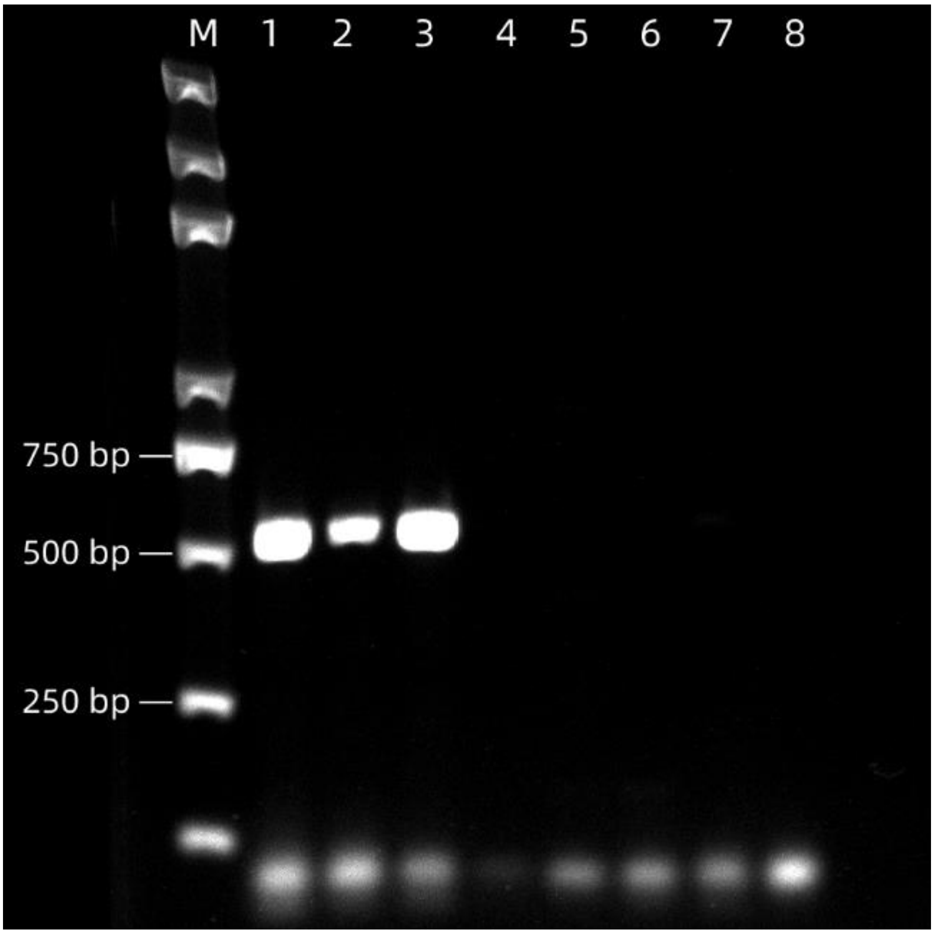

Figure S1. Detection of *A. apis* in the gut sample from *A. apis*-infected larvae and controls by PCR test at 3 days post-infection. The gut samples (lanes 1-3) from the *A. apis*-infected larvae were positive for *A. apis* infection (550 bp), and the gut samples (lanes 5-7) from the control larvae were negative for *A. apis* infection. M indicates DNA markers; lanes 4 and 8: negative controls (no template) for guts samples of *A. apis*-infected larvae and controls respectively.
