# Supplementary material for: Changes in Antioxidant Enzymes Activity and Metabolomic Profiles in the Guts of Honey Bee (Apis mellifera) Larvae Infected with Ascosphaera apis
Source: Insects. 2020 Jul 6;11(7):419. doi: 10.3390/insects11070419 (PMC7412215; doi:10.3390/insects11070419)
Supplement: Supplementary file 1 [file insects-11-00419-s001.zip › Supplementary Files/Figure S2.pdf]

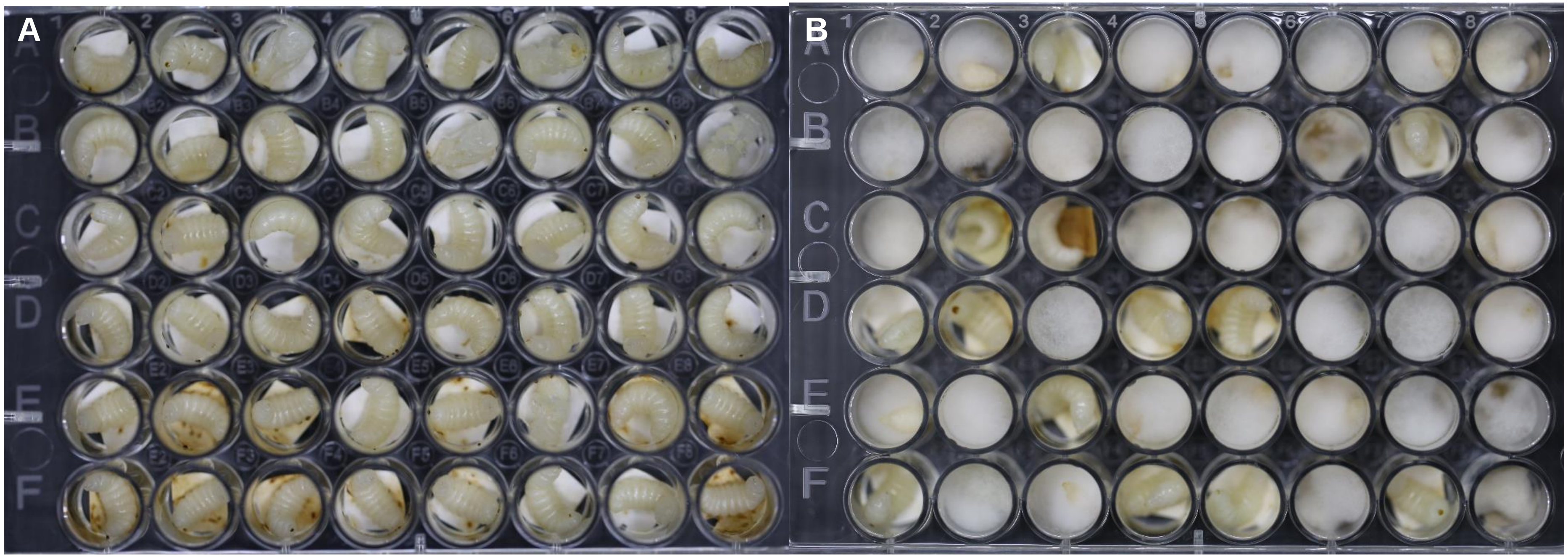

Figure S2. All the control larvae developed well without any obvious disease symptoms (A); the *Apis*-infected larvae developed typical chalkbrood symptoms at 6 days post-infection (B), and the percentage of larvae covered with white, cotton-like mycelium was 77% (37/48) in this 48-well microtiter plate.
