# Supplementary material for: Changes in Antioxidant Enzymes Activity and Metabolomic Profiles in the Guts of Honey Bee (Apis mellifera) Larvae Infected with Ascosphaera apis
Source: Insects. 2020 Jul 6;11(7):419. doi: 10.3390/insects11070419 (PMC7412215; doi:10.3390/insects11070419)
Supplement: Supplementary file 1 [file insects-11-00419-s001.zip › Supplementary Files/Table S2.docx]

**Table S2.** The six most relevant pathways were selected based on –ln P-value > 1 and pathway impact score > 0.01.

| **Pathway** | **Mapped significantly differential metabolites** | **-ln(p)** | **Impact** |
| --- | --- | --- | --- |
| Phenylalanine, tyrosine and tryptophan biosynthesis | L-Tyrosine | 1.90 | 0.50 |
| Taurine and hypotaurine metabolism | Taurine | 1.54 | 0.50 |
| Pentose phosphate pathway | D-Ribose 5-phosphate, D-Ribose, D-Ribulose 5-phosphate, D-Erythrose 4-phosphate | 5.25 | 0.34 |
| Tyrosine metabolism | L-Tyrosine, Gentisaldehyde | 1.10 | 0.19 |
| Galactose metabolism | Melibiose, alpha-D-Galactose 1-phosphate | 1.30 | 0.17 |
| Purine metabolism | D-Ribose 5-phosphate, Inosinic acid, Uric acid  2'-Deoxyadenosine 5'-monophosphate (dAMP) | 1.42 | 0.13 |
